# Adrenaline May Contribute to Prothrombotic Condition via Augmentation of Platelet Procoagulant Response, Enhancement of Fibrin Formation, and Attenuation of Fibrinolysis

**DOI:** 10.3389/fphys.2021.657881

**Published:** 2021-05-05

**Authors:** Agata Golaszewska, Tomasz Misztal, Natalia Marcinczyk, Ewa Chabielska, Tomasz Rusak

**Affiliations:** ^1^Department of Physical Chemistry, Medical University of Bialystok, Bialystok, Poland; ^2^Department of Biopharmacy, Medical University of Bialystok, Bialystok, Poland

**Keywords:** platelet, thrombus formation, procoagulant activities, phosphatidylserine exposure, adrenaline, fibrinolysis

## Abstract

**Background:** Adrenaline is believed to play a role in thrombosis and hemostasis. The complex effect of its clinically relevant concentrations on thrombus formation, coagulation and fibrinolysis in human blood has never been specifically studied.

**Methods:** Confocal microscopy was used to study thrombus formation under flow, exposure of phosphatidylserine (PS) in adhered platelets, to evaluate clots density, and to measure kinetics of fibrin formation and external fibrinolysis under flow. Flow cytometry was utilized to assess PS exposure in non-adhered platelets. Kinetics of clot formation and internal fibrinolysis was evaluated by thromboelastometry. Platelet aggregation was measured by optical aggremometry. Kinetics of clot retraction was assessed by using digital camera.

**Results:** We found that adrenaline (1–10 nM) is able to enhance platelet activation evoked by subthreshold collagen (150 ng/ml), resulting in augmentation of platelet aggregation, thrombus formation under arterial flow conditions, platelet PS exposure, and formation of platelet-fibrin clots. The development of platelet procoagulant response evoked by adrenaline + low collagen was associated with the formation of denser platelet-fibrin clots and the decrease in rate of fibrinolysis despite whether lysis was initiated inside (internal fibrinolysis) or outside the clot (external fibrinolysis). The above phenomena were abolished by the α_2_-adrenergic receptor antagonist, rauwolscine. Adrenaline-collagen synergism, expressed as PS exposure, was significantly reduced by cyclooxygenase inhibitor (acetylsalicic acid), GPIIb/IIIa receptor blocker (tirofiban), and P2Y_12_ receptor antagonist (PSB 0739).

**Conclusion:** Clinically relevant concentrations of adrenaline may significantly augment responses of human platelets in the presence of subthreshold concentrations of collagen, which should be considered during therapies involving adrenaline infusion. Routinely used antiplatelet drugs may reduce the prothrombotic state evoked by adrenaline-collagen synergism.

## Introduction

Adrenaline (epinephrine) is a catecholamine hormone engaged in a broad spectrum of human physiology, released into the bloodstream after the stimulation of sympathetic nervous system (Goldstein, 2010). The presence of adrenaline in blood may also be a result of clinical intervention during cardiac arrest (Mentzelopoulos et al., 2013) and anaphylactic shock (Sheikh et al., 2009; Ring et al., 2018). The concentration of adrenaline in human blood has been estimated to vary from below 1 nM up to 20 nM during severe stress reaction (Dimsdale and Moss, 1980; Seiss et al., 1982; Ardlie et al., 1985). Numerous clinical observations point to a positive correlation between stress and thrombotic tendencies (reviewed in Bentur et al., 2018). Initially, it was believed that the impact of adrenaline on hemostasis was associated predominantly with its vasoconstrictor properties (von Känel, 2015). Further studies revealed that human platelets express α_2A_-adrenergic receptors (AR), coupled with G_z_ protein, a subtype of G_i_ protein (Mukherjee, 1981; Yang et al., 2000; Woulfe et al., 2002). Binding adrenaline to platelet α_2_-AR receptors results in the inhibition of adenylate cyclase (AC) and, hence, in a decrease of cytosolic cyclic AMP concentration (negative regulator of platelet activation; Sachs and Nieswandt, 2007; Stalker et al., 2012). Therefore, adrenaline may facilitate platelet activation process and thus support thrombus formation. The research also points to the involvement of Na^+^/H^+^ exchanger (Steen et al., 1989), arachidonic acid cascade (Cameron and Ardlie, 1982), and PI-3K kinase (Selheim et al., 2000) in adrenaline-evoked platelet activation. However, adrenaline *per se* appears to trigger platelet aggregation *in vitro* only at supraphysiological (micromolar) concentrations (Shattil et al., 1989; Spalding et al., 1998; Singh et al., 2018). Conversely, nanomolar concentrations of adrenaline have been found to enhance platelet adhesion (Mustonen and Lassila, 1996; Eriksson and Whiss, 2013), aggregation (Mills and Roberts, 1967; Cameron and Ardlie, 1982; Wagner et al., 1996), and secretion (Singh et al., 2018) evoked by low concentrations of principal platelet agonists.

In 1997 (Nakamura et al., 1997) demonstrated for the first time that adrenaline, at the concentration close to a clinically relevant range (20 nM), is capable of inducing platelet microaggregates formation. However, the relevance of adrenaline-platelet interaction in normal hemostasis had been unknown for a long time until a few research groups observed a moderately prolonged bleeding time and an impaired thrombi formation in α_2_-AR deficient patients (Tamponi et al., 1987; Rao et al., 1988; Lindkvist et al., 2019). These observations suggested a non-redundant role of adrenaline in normal hemostatic response.

More recently, experimental models employing knockout animals as examination tools have provided new essential information on the role of adrenaline in thrombosis and hemostasis. Pozgajová et al. (2006) reported a highly variable effect of α_2_-AR deficiency on tail bleeding time in mice and a mild effect on thrombus stability in 2 separate thrombosis models, suggesting the context-depending role of adrenaline in platelet signaling and hemostasis. Shen et al. (2017) revealed that the ablation of G_z_ protein did not impair thrombus formation in the laser-induced mice thrombosis model. However this study did not include elevated blood adrenaline while local generation of thrombin at a site of injury—in this particular model—is relatively high (Furie and Furie, 2008; Welsh et al., 2014; Shen et al., 2017). Since thrombin is a potent platelet activator, the engagement of a weak agonist, i.e., adrenaline, was most likely shrouded. On the other hand, mice lacking α_2_-AR, as well as mice deficient in G_z_ protein, demonstrated resistance to lethal thromboembolism induced by the injection of collagen and adrenaline combination (Yang et al., 2000). Since collagen/adrenaline-induced thromboembolism is associated with emboli rich in both platelets and fibrin (Konstantinides et al., 2004) one may hypothesize that the hormone can contribute not only to the augmentation of platelet aggregation but also to the enhancement of platelet-related procoagulant response and the thrombin generation associated with it. Such a role of adrenaline has never been considered in humans.

Consequently, this study was designed to systematically examine, for the first time, the impact of clinically relevant adrenaline concentrations (1–10 nM), alone or in combination in the subthreshold concentration of collagen, at early and late stages of hemostatic response: thrombus formation, procoagulant response, platelet-fibrin clot formation, its retraction and lysis in human blood. It is worth emphasizing that most experiments were performed under flow conditions close to those which are present in arteries.

## Materials and Methods

### Materials

Hepes, apyrase, adrenaline [(-)-epinephrine (+)-bitartrate salt)], ADP disodium salt, human fibrinogen, acetylsalicic acid (ASA), bovine serum albumin (BSA) were from Sigma (Merck). Type I collagen was from Chrono-log. Batroxobin (Dade Behring) and tissue factor (Innovin) were from Dade Behring. DiO (3-octadecyl-2-[3-(3-octadecyl-2(3H)-benzoxazolylidene)-1 propenyl]-, perchlorate, AlexaFluor (AF)488-human fibrinogen and AF647-annexin V were from Thermo Fisher. Phycoerythrin (PE)-anti human CD41a antibody and FITC-annexin V were from Becton Dickinson. Recombinant tissue plasminogen activator (Actilyse, Boehringer Ingelheim). Rauwolscine hydrochloride and PSB 0739 were from Tocris. Tirofiban (Aggrastat) was from Merck. Nadroparinum calcinum (Fraxiparine) was from Glaxo Smith Kline). PPACK hydrochloride was from Santa Cruz Biotech. Other chemicals were from Sigma (Merck). Throughout the text “rauwolscine” means a water solution of rauwolscine hydrochloride.

### Blood Collection and Preparation

Venous blood was collected from healthy volunteers with minimum trauma and stasis via a 21-gauge needle (0.8 × 40 mm) into 10 ml polypropylene tubes containing 1 ml of 130 mM trisodium citrate. All procedures were conducted in accordance with the principles of Declaration of Helsinki and the study was approved by the local Ethics Committee on human research (R-I-002/337/2017, R-I-002/307/2018). Platelet-rich plasma (PRP) was obtained by centrifugation of whole blood at 200 × g for 20 min. Preparation of washed platelets was performed as essentially described in Misztal et al. (2015).

### Confocal Microscopy Imaging

For flow chamber experiments and clot structure imaging we used fixed-stage microscope Zeiss Axio Examiner Z.1 (Carl Zeiss Microscopy GmbH, Germany) equipped with W Plan-Apochromat 20 × /1.0 water immersion objective (Carl Zeiss Microscopy GmbH, Germany). During the experiment AF488-fibrinogen, DiO were excited by 488 nm laser (LaserStack 488 nm, 3iL33, Intelligent Imaging Innovations, Inc., United States) while AF647-annexin V were excited by 640 nm laser from the same manufacturer. Pictures were recorded with Confocal Scanner Unit CSU-X1 (Yokogawa Electric Corporation, Japan) in one focal plane (2D imaging). Collected images were analyzed using SlideBook 6 (Intelligent Imaging Innovations, Inc., United States).

### Platelet Aggregation

Platelet aggregation in PRP was measured optically as increase of light transmittance, associated with aggregation progress (Born and Cross, 1963) using Elvi Logos 840 (Milano, Italy) aggregometer connected with personal computer via e-corder 401 (eDAQ Pty Ltd., Denistone East, Australia). Aggregation was monitored using eDAQ Chart software.

### Flow Studies

### Investigation of Platelet Adhesion to Collagen, and Assessment of the Procoagulant Index

Degreased, collagen (type I, 50 μg/ml) microspots-coated coverslips were mounted onto a transparent, polycarbonate-made parallel-plate flow chamber (50 μm in depth, 3 mm in width, and 30 mm in length (Zwaginga et al., 2006a,b; Van Kruchten et al., 2012; Sakariassen et al., 2015; Supplementary Figure 1) and pre-rinsed with Hepes buffer (pH 7.45) containing 0.1% BSA, as essentially described in de Witt et al. (2014). Before use, coverslips were blocked by BSA solution (1% w/v) in above Hepes buffer to prevent activation of contact-dependent coagulation pathway and to diminish contact of platelets with glass surface. Freshly collected trisodium citrate-anticoagulated whole blood samples were immediately supplemented with Fraxiparine (1 U/ml) and PPACK (40 μM, to inhibit factor Xa and thrombin activity) and incubated without (control) or with adrenaline (1–10 nM) for 2 min. In some experiments, before addition of adrenaline, samples were incubated with rauwolscine (10 μM, for 10 min). Next, the samples (500 μl) were preincubated for 2 min with DiO (1 μM, to visualize platelet plasma membrane), followed by the supplementation with 1,5 μl of 1 M MgCl_2_ and CaCl_2_ solution (hence the final concentration of added MgCl_2_/CaCl_2_ in samples was 3 mM) just before being perfused through the flow chamber for a time period sufficient for full-thrombus formation on collagen spots (4 min at shear rate 1,000 s^–1^ or 2.5 min at 1,600 s^–1^). Thrombi formed during the blood flow were post-stained by a 2-min perfusion (1,000 s^–1^) with AF647-annexinV (5 μg/ml) in Hepes buffer pH (7.45) supplemented with 0.1% BSA. After staining (under stasis, typically 2 min), the unbound label was removed by a short perfusion with the same Hepes buffer without labeled proteins. No fixative was used. End-stage fluorescence images were generated using a confocal laser scanning microscope. The surface coverage by adhered platelets in the area of 4,800 μm^2^ was calculated with the use of ImageJ free software (Rasband, 1997–2016). The procoagulant index was calculated as a surface area of phosphatidylserine (annexin V)-positive platelets divided by the surface area of all adhered platelets from the considered location (Marcinczyk et al., 2020). For analysis of thrombus volume changes under flow thrombi were formed as described above, and images were captured in real time using a confocal microscope, with 1-μm sections acquired in 1-min intervals up to 5 min. Analysis of thrombus volume was performed using SlideBook 6 software.

### Measurements of Real-Time Fibrin Formation and External Fibrinolysis

Whole blood samples (without any thrombin inhibitors), prepared in the same manner as in the above paragraph, were preincubated for 2 min with AF488-fibrinogen (to visualize fibrin formation) and supplemented with MgCl_2_ (3 mM) and CaCl_2_ (0.75 mM) just before being perfused through the flow chamber for 4 min at a shear rate 1,000 s^–1^ over the collagen-coated microspots. Thrombi formed during blood flow were post-stained by 2 min perfusion (1,000 s^–1^) with AF647-annexinV (5 μg/ml) in Hepes buffer (pH 7.45) supplemented with 0.1% BSA after which annexin V-free Hepes buffer was continuously perfused at shear rate 1,000 s^–1^. Real-time formation of AF488-fibrin(ogen) was recorded in a confocal microscope. The final concentration of CaCl_2_ used in fibrin formation under flow experiments was relatively low compared to the routinely used protocol (Van Kruchten et al., 2012). This particular concentration of CaCl_2_ was selected during preliminary experiments in a way to ensure that differences between studied samples can be measureable. Considering strongly procoagulant conditions present within samples, i.e., platelets activated by fibrillar collagen or by combination of adrenaline plus collagen (plus transient pool of the secreted secondary agonists, and also secreted Ca^2+^) such relatively low CaCl_2_ concentration allowed to obtain fibrin formation velocity suitable to detect differences between studied samples. Kinetics of fibrin formation was recorded as a function of fibrin(ogen)-related fluorescence increase in time. Furthermore, pictures reflecting the progress of annexin V binding (associated with the 10, 50, and 100% of annexin V fluorescence appearance) to PS-exposing platelets were collected. After the formation of fibrin, samples were washed with Hepes buffer containing tPA (40 nM) to assess external fibrinolysis (associated with binding and activation of fibrinolytic factors at the clot surface and their diffusion across fibrin mesh; Tutwiler et al., 2019). A temporal decrease of fibrin-related fluorescent signal (reflecting progress of fibrinolysis) was recorded until fluorescence reached the background level. We observed and recorded the entire process in real time. No fixative was used.

### Measurements of the Kinetics of Clot Retraction in the Absence of Thrombin

The measurements of clot retraction were performed in a system containing purified fibrinogen and washed platelet. To avoid activation of contact-dependent coagulation pathway in possible traces of plasma in washed platelets suspension (and hence thrombin generation), experiments were conducted in plastic tubes. In brief, aliquots (0.5 ml) of washed platelets were preincubated without (control) or with adrenaline (1–3 nM) for 2 min at 37°C followed by the transfer of 0.2 ml of such prepared samples into tubes containing 1.55 ml of Tyrode-Hepes buffer (pH 7.45) supplemented with human fibrinogen (2 mg/ml), CaCl_2_ (2.5 mM) and ADP (2 μM, i.e., concentration of ADP likely to be present within thrombus core (Flamm et al., 2012)–to stimulate platelet contractile apparatus). Coagulation was triggered by the addition of batroxobin [5 batroxobin units (BU)/ml final conc.] which induces the formation of fibrin without platelet activation (De Clerck et al., 1975). The pictures of clots ongoing retraction were taken in the course of 1 h at 10 min intervals using a CCD camera. The recorded decrease in clots volume was quantified by the assessment of the clot area with the use of Motic Images Plus 2.0 ML software, and the data were processed using GraphPad Prism 5. Clot surface areas were plotted as a percentage of maximal retraction at specific time points. The data was expressed as follows: percentage of retraction = ((area *t*_0_-area *t*)/area *t*_0_) × 100% (Osdoit and Rosa, 2001).

### Cytometric Measurements of Platelet PS Exposure

For phosphatidylserine (PS) exposure measurements we used PRP to exclude the erythrocytes (RBC) lysis step which may in turn enrich samples with RBC-originated ADP and provoke additional platelet activation. PRP samples were incubated without any additions (control) or with rauwolscine (10 μM, for 10 min) followed by the addition of adrenaline (1–10 nM), collagen (150/ml) or a combination of both agonists (adrenaline was added first, 1 min before collagen) with initial stirring (30 s) under the control of an aggregometer (to avoid unnecessary platelet aggregation). After 15 min of incubation at room temperature PS exposure was assessed by flow cytometry, as previously described in Misztal et al. (2015) with the exception of using annexin V—instead of lactadherin—as a probe for PS. After final dilution with Hepes buffer (pH 7.45) containing saturating CaCl_2_ concentration (2 mM), samples were incubated with fluorescently labeled anti-CD41a antibody (probe for platelets) and annexin V (probe for exposed PS). In additional experiments, PRP samples were preincubated (for 10 min) with cyclooxygenase inhibitor (ASA, 200 μM), GPIIb/IIIa receptor blocker (tirofiban, 500 nM) or P2Y_12_ receptor antagonist (PSB-0739, 100 μM) before the addition of adrenaline and collagen combination. In additional set of experiments adrenaline was added at 10 μM concentration to evoke comparable PS exposure as combination of nanomolar adrenaline + collagen.

### Thromboelastometric Measurements of the Kinetics of Platelet-Fibrin Clot Formation and Internal Fibrinolysis

Thromboelastometric measurements were performed using ROTEM^®^ Delta rotational thromboelastometric system (Tem International GmbH, Manheim, Germany) (Luddington, 2005). Whole blood samples were incubated at room temperature without any addition (control) or with rauwolscine (10 μM, for 10 min), followed by the addition of adrenaline (1–10 nM) and collagen (Chrono-Par reagent, 150 ng/ml, added 2 min after adrenaline) with initial stirring (30 s). After 10 min of incubation the samples were analyzed toward the kinetics of clot formation. All ROTEM coagulation measurements were performed by the same experienced operator as follows: 320 μl of sample was transferred into a preheated (37°C) cup containing 20 μl of 0.2 M calcium chloride as an activator and repeatedly gently pipetted to mix the components. We measured the parameters characterizing clotting initiation (CT, clotting time; CFT, clot formation time), propagation (alpha angle; MaxV, maximal velocity of clot formation; MaxV-t, time to reach maximal velocity of clotting), and stabilization (MCF, maximum clot firmness; G, shear elastic modulus strength).

We used thromboelastometric approach to examine internal fibrinolysis, associated with fibrin digestion inside the clot (Tutwiler et al., 2019). To assess the kinetics of fibrinolysis, whole blood samples, prepared as above, were mixed with tissue factor (Innovin, 100 ng/ml) and recombinant tissue plasminogen activator (Actilyse, 120 ng/ml) and lysis onset time (LOT) was measured (Misztal et al., 2019).

### Confocal Analyses of Clot Structure

PRP samples were preincubated at 37°C addition (control), with or without rauwolscine (10 μM, for 10 min), followed by the addition of adrenaline (1–10 nM) and collagen (150 ng/ml, added 2 min after adrenaline) with initial stirring (30 s). After 15 min of incubation, AF488-fibrinogen was added to all samples, the clotting was triggered by recalcification (by the addition of CaCl_2_ to the final concentration of 18 mM in each sample) and after 2h-long incubation in dark, humid chamber (at 37°C) the resulting platelet-fibrin clots were analyzed with the use the confocal microscope toward relative fibrin density. The preparation and visualization of platelet-fibrin clots with the use of confocal microscope was performed as essentially described in Gromotowicz-Poplawska et al. (2019). The samples were examined after the transfer to microchamber slides (Ibidi μ-slide VI; Animalab). At least 10 pictures of different areas of each clot were taken. Relative clots density was established as a number of fibers crossing a single, randomly placed 30 μm-long straight line. At least 10 measures from each picture (5–8 pictures selected from each single experiment) were performed.

### Data Analyses

Data were evaluated using GraphPad Prism 5 (GraphPad Software). The Shapiro–Wilk test was performed to determine the normal distribution. The differences between two groups were assessed by means of the Mann–Whitney *U*-test. In experiments where multiple comparisons were considered, Kruskal-Wallis test was used. The data are shown as the mean (± S.D.)—in case of normal distribution—or as the median (interquartile range)—for non-normal distributed data sets—of the number of determinations (n) or as a percentage compared to the control. In all experiments a *p* < 0.05 was considered to be significant.

## Results

### Augmentation of Platelet Aggregation, Adhesion, and Clot Retraction by Adrenaline

**FIGURE 1:**
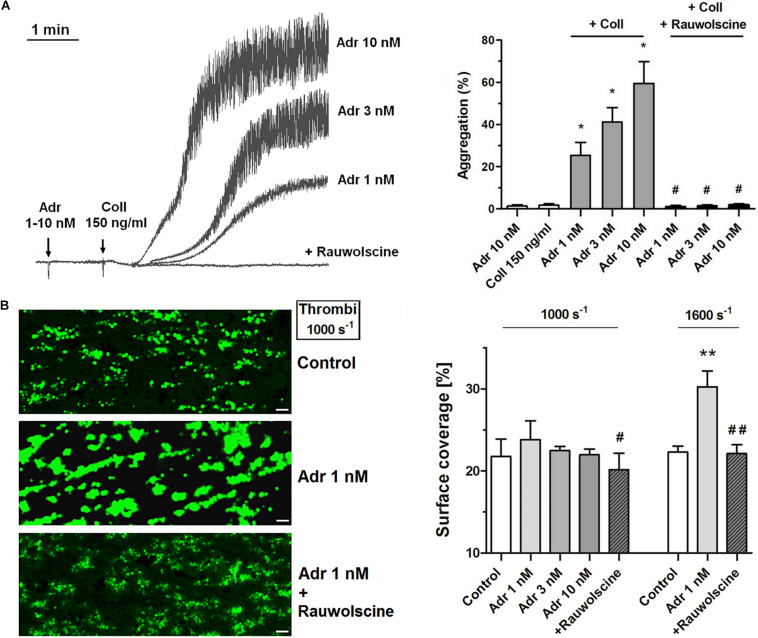
Effect of nanomolar adrenaline concentrations on platelet aggregation and thrombus formation under flow **(A)**. Samples of PRP were incubated without or with rauwolscine (10 μM, for 10 min) and supplemented with adrenaline (1–10 nM), collagen (150 ng/ml) or combination of both agonists (in that case adrenaline was added first, 1 min before collagen). Representative aggregation curves from one (out of 6) experiment (one experiment is considered as aggregation measurements—at least in three repetitions—using PRP obtained from one donor) and aggregation extent are presented in **(A)**. Aggregation obtained in the presence of 5 μg/ml of collagen was considered as maximal aggregation. Adrenaline concentrations below 10 nM also did not trigger platelet aggregation (not shown). Data are means ± S.D. from *n* = 6 experiments. *p < 0.05 vs. “Coll 150 ng/ml.” ^#^*p* < 0.05 vs. sample containing collagen (150 ng/ml) and appropriate concentration of adrenaline. **(B)** PPACK-anticoagulated whole blood samples (supplemented with DiO to visualize platelets) were incubated without any addition (control) or with adrenaline (for 2 min) or rauwolscine (10 μM, for 10 min) + adrenaline (for 2 min). Next, samples were perfused over collagen-coated surfaces at shear rate 1,000 or 1,600 s^– 1^ to form thrombi. Surface coverage area was calculated from end-stage confocal pictures. Data are means ± S.D. from *n* = 9 experiments. **p < 0.01 vs. control; ^#^*p* < 0.05, ^##^*p* < 0.01 vs. adrenaline 1 nM. Presented pictures are representative for experiments conducted at shear rate 1,000 s^– 1^.

Adrenaline (1–10 nM) in combination with the subthreshold concentration of collagen (150 ng/ml) evoked platelet aggregation. This phenomenon was abolished by the preincubation of samples with adrenergic receptor antagonist, rauwolscine (Figure 1A). Studied concentration of adrenaline (1–10 nM) or collagen alone did not evoke microaggregates formation measured by electronic counting method (as a potential decrease in PLT count due to microaggregates formation) and by flow cytometry (lack of potential CD41a-positive objects with elevated forward scatter, i.e., aggregates, after adrenaline (or collagen) treatment.

To investigate whether adrenaline may modulate platelet adhesion in a shear-dependent manner, we recorded platelet accumulation on collagen under flow conditions corresponding with two arterial shear rates (1,000 and 1,600 s^–1^). The preincubation of whole blood samples with adrenaline (1–10 nM) did not affect the coverage of collagen-coated surfaces by thrombi under share rate 1,000 s^–1^. At a shear rate 1,600 s^–1^ adrenaline (1 nM) enhanced platelet adhesion to collagen (manifested as a higher degree of surface coverage); this effect was reduced by rauwolscine (Figure 1B).

**FIGURE 2:**
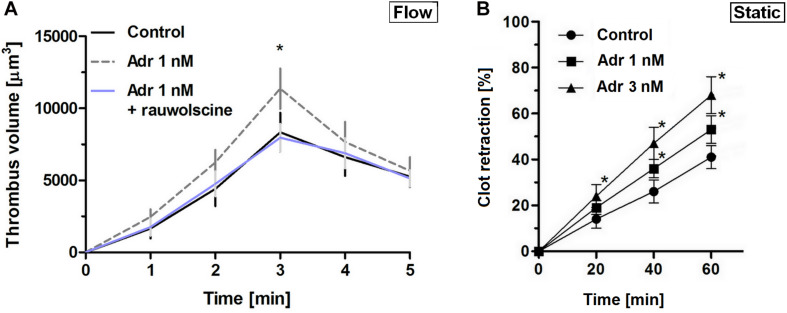
Effect of nanomolar adrenaline concentrations on clot retraction. **(A)** To quantify thrombus volume, whole blood, preincubated with adrenaline, with rauwolscine + adrenaline or without any additions (control) was perfused over collagen at shear rate of 1,000 s^– 1^ and z-stacks were captured in real-time using a confocal microscope. Changes in thrombus volume was quantified using SlideBook 6 software. Data are means ± S.D. from *n* = 9 experiments. *p < 0.05 vs. control. **(B)** Samples of washed platelets were preincubated without (control) or with adrenaline (for 2 min) followed by a transfer to Tyrode’s buffer containing CaCl_2_ (2.5 mM) and ADP (2 μM, to stimulate platelet contractility) and batroxobin (5 BU/ml, to evoke coagulation without additional platelet activation). Decrease in clots volume was recorded by digital camera in 10 min intervals up to 60 min and expressed as clot retraction%. No retraction was noticed in the absence of ADP and adrenaline in the above experimental system (not shown). Data are means ± S.D. from *n* = 3 experiments. *p < 0.05 vs. control.

To verify whether lack of adrenaline effect on adhesion at lower shear may be associated with the enhancement of platelet contractility (and hence in a stronger thrombi consolidation, potentially masking higher extent of platelet adhesion), we assessed contraction course of thrombi under flow and static conditions. In that second case, batroxobin-derived platelet-fibrin clots were formed in the absence or presence of adrenaline and ADP (the major thrombi-originated platelet agonist). The rate of contraction of such clots was markedly higher in adrenaline-enriched samples under both conditions (Figures 2A,B).

### Adrenaline-Related Enhancement of Platelet PS Exposure and Coagulation Kinetics

**FIGURE 3:**
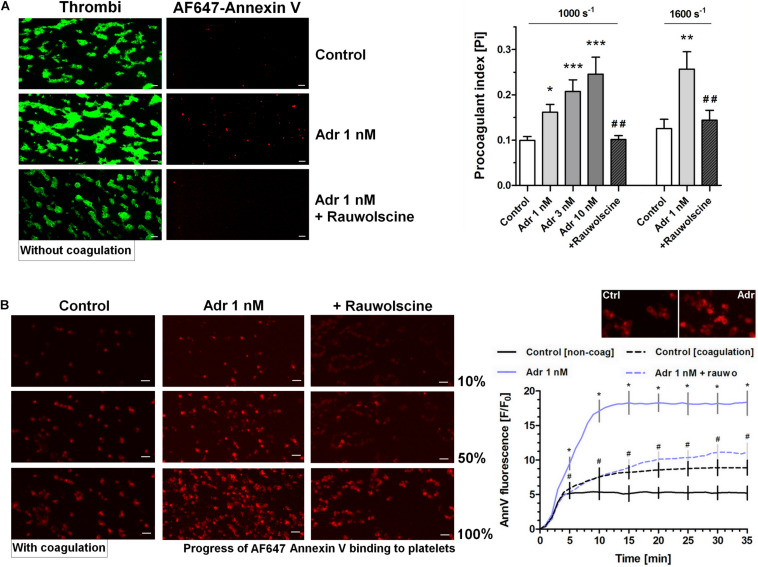
Effect of adrenaline on platelet PS exposure under flow in the absence or presence of thrombin. **(A)** PPACK-anticoagulated whole blood samples (supplemented with DiO to visualize platelets) were incubated without any addition (control) or with adrenaline (for 2 min) or rauwolscine (10 μM, for 10 min) + adrenaline (for 2 min). Next, samples were perfused over collagen-coated surfaces at shear rate 1,000 or 1,600 s^– 1^ to form thrombi. Thrombi were post-stained with AF647 annexin V to visualize platelet membrane-exposed phosphatidylserine (PS). Ratio of annexin-related fluorescence to DiO fluorescence, i.e., procoagulant index, was calculated from end-stage confocal pictures. Data are means ± S.D. from 9 experiments. *p < 0.05, **p < 0.01, and ***p < 0.001 vs. control; ^##^*p* < 0.01 vs. adrenaline 1 nM. Presented pictures are representative for experiments conducted at shear rate 1,600 s^– 1^. **(B)** Whole blood samples (without any direct thrombin inhibitor) were incubated without any addition (control) or with adrenaline (for 2 min) or rauwolscine (10 μM, for 10 min) + adrenaline (for 2 min). Next, samples were perfused over collagen-coated surfaces at shear rate 1,000 s^– 1^ to form thrombi. No significant differences in surface area coverage by platelets between conditions with and without coagulation were observed. Afterward, thrombi were washed with AF647 annexin V and progress of its accumulation on the surface of PS-exposed platelets was recorded using confocal microscope. Representative pictures reflecting progress of annexin V binding (associated with 10, 50, and 100% of total annexin V fluorescence in appropriate sample) are presented (*n* = 4). Typical picture of balloon platelets formed in the absence (Ctrl) or in presence of 1 nM adrenaline (Adr) is shown as the insert in chart. For kinetics measurements, raw fluorescence intensities from time-series records (measurements in 1 min intervals) were quantified after regions of interest were chosen and images corrected for background noise. For each record, relative fluorescence (*F*/*F*_0_) is reported, where *F*_0_ designates the background-subtracted fluorescence level before platelet activation. Data are expressed as means ± S.D. from 4 experiments. *p < 0.05 vs. control; ^#^*p* < 0.05 vs. adrenaline 1 nM. In panel B, *p < 0.05 vs. control (with coagulation). In all panels white bar = 10 μm.

To examine whether adrenaline is able to enhance GPVI-dependent platelet procoagulant response (and the potential shear-dependency of this phenomenon), we performed measurements of phosphatidylserine (PS) exposure within thrombi formed on collagen under arterial flow conditions (shear 1,000 and 1,600 s^–1^). As can be seen (in the absence of thrombin activity) adrenaline (1–10 nM) produced a significant increase in the platelet procoagulant index, translating into a higher content of PS-exposing platelets subpopulation within thrombi. The adrenaline effect was strongly reduced by rauwolscine (Figure 3A). We also conducted thrombus formation under flow experiments without any thrombin inhibitors to evaluate whether local activity of *in situ* generated thrombin may modulate the dynamics of platelet PS exposure in the presence of adrenaline. In such conditions the amount of platelet-bound annexin V (probe for exposed PS) gradually increased in time. Adrenaline (1 nM) enhanced the total number of PS-positive platelets [especially balloon platelets (insert in the chart)] and the rate of their appearance. The presence of rauwolscine prevented from these effects (Figure 3B).

**FIGURE 4:**
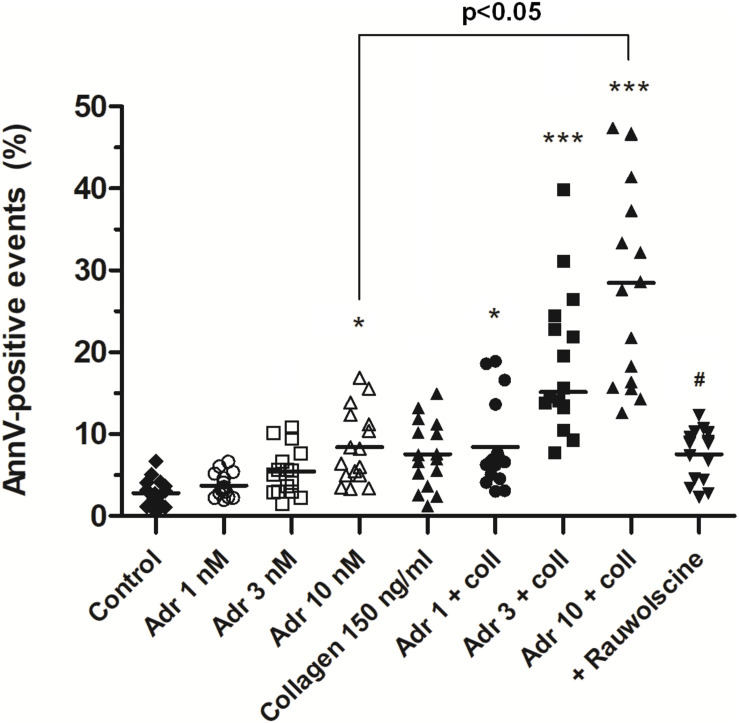
Effect of adrenaline on PS exposure in platelet suspension. PRP samples were incubated without any additions (control), with or without rauwolscine (10 μM, for 10 min) followed by the addition of adrenaline (1–10 nM), collagen (150 ng/ml) or combination of both agonists with initial stirring (30 s). After incubation (15 min at room temperature), samples were supplemented with appropriate fluorescent probes, and after final dilution analyzed toward PS exposure by flow cytometry (*n* = 16). *p < 0.05, ***p < 0.001 vs. control; ^#^*p* < 0.001 vs. adrenaline 10 nM + coll.

In order to check whether adrenaline is capable to evoke platelet PS exposure also in non-adhered platelets (platelet suspended in plasma), we performed flow cytometry measurements. The incubation of PRP samples with adrenaline (10 nM) resulted in a significant increase of PS-exposed platelet population. This effect was notably enhanced by the coincubation of adrenaline (1–10 nM) with subthreshold concentration of collagen (150 ng/ml) and was markedly reduced by rauwolscine (Figure 4).

**FIGURE 5:**
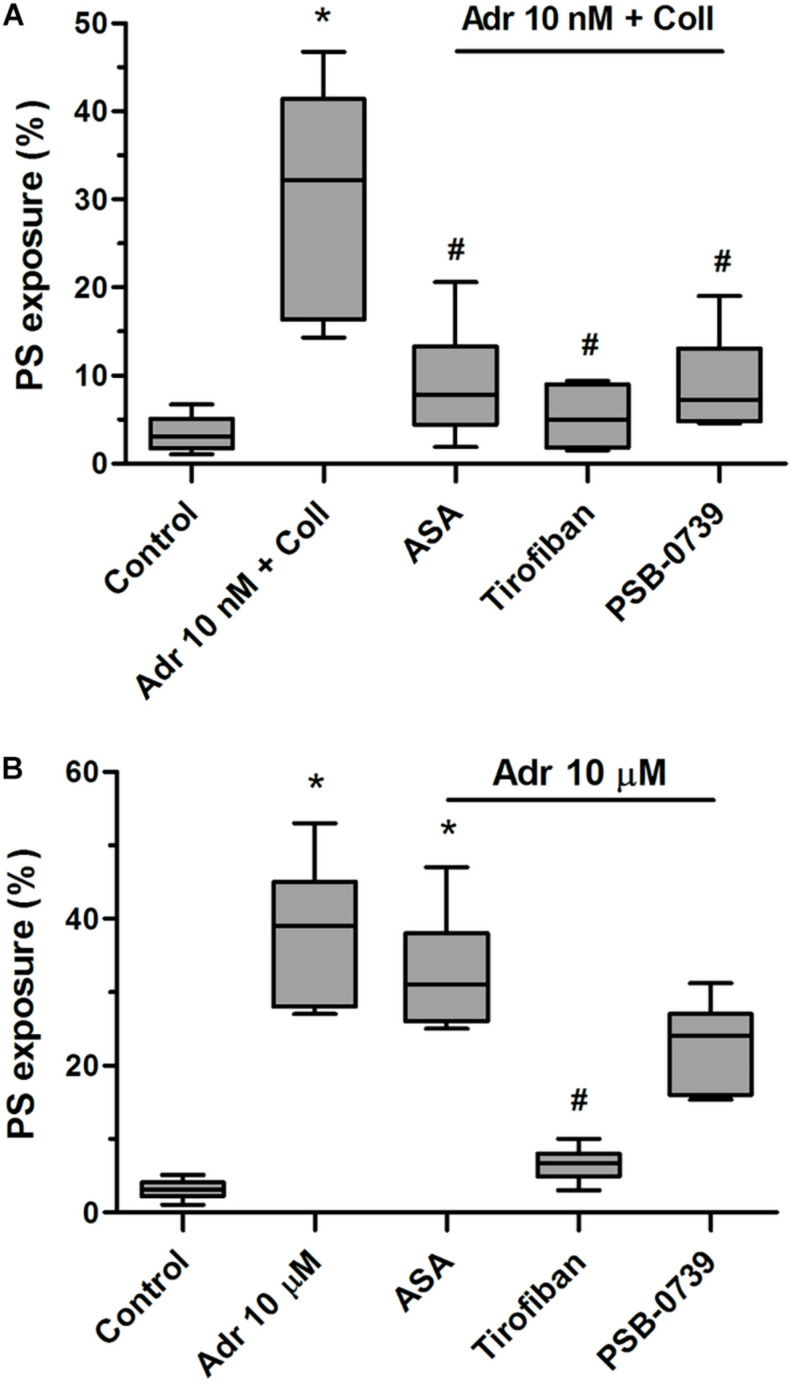
Reduction of adrenaline and collagen-evoked platelet PS exposure by cyclooxygenase inhibitor, GPIIb/IIIa blocker, and P2Y_12_ receptor antagonist. Samples of PRP were incubated for 10 min with cyclooxygenase inhibitor (ASA, 200 μM), GPIIb/IIIa blocker (tirofiban, 500 nM), and P2Y_12_ receptor antagonist (PSB-0739, 100 μM). Next, samples were supplemented with adrenaline (10 nM) and collagen (150 ng/ml, **A**) or with adrenaline alone (10 μM, **B**). Samples were incubated at room temperature for 30 min. Attention was taken to do not allow platelets to form aggregates. Control samples were without any addition. After incubation, samples were supplemented with appropriate fluorescent probes, and after final dilution analyzed toward PS exposure by flow cytometry. Presented results are medians with interquartile range from 6 independent experiments. *p < 0.05 vs. control in both panels; ^#^*p* < 0.05 vs. Adr 10 nM + coll in **(A)** and *p* < 0.05 vs. Adr 10 μM in **(B)**.

To check what signaling events are crucial for PS exposure evoked by adrenaline and its combination with collagen we performed experiments with cyclooxygenase inhibitor (ASA), P2Y_12_ receptor antagonist (PSB-0739), and GPIIb/IIIa blocker (tirofiban). As is seen in Figure 5A, PS exposure triggered by adrenaline (10 nM) + collagen (150 ng/ml) combination was strongly reduced by the preincubation of samples with ASA (200 μM), PSB-0739 (100 μM, more than 70% of inhibition in both cases), and tirofiban (500 nM, more than 90% of inhibition). When platelets were stimulated with micromolar (10 μM) adrenaline *per se*, to obtain similar degree of PS exposure as in case of nanomolar adrenaline + collagen condition, only tirofiban reduced this effect significantly (Figure 5B) suggesting the involvement of different signaling pathways than in case of adrenaline + collagen synergism.

**FIGURE 6:**
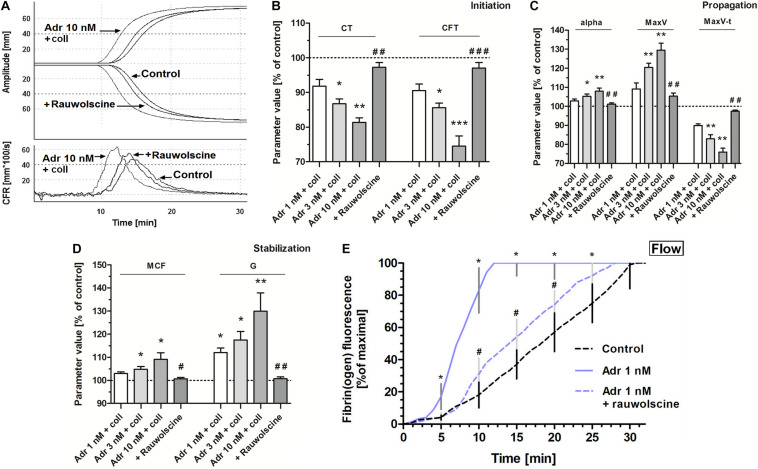
Effect of adrenaline on the kinetics of platelet-fibrin clot formation. Whole blood samples were incubated with or without rauwolscine (10 μM, for 10 min) followed by the addition of adrenaline (for 2 min) and collagen (150 ng/ml) with initial stirring (30 s). After 10 min of incubation, at room temperature, samples were analyzed toward kinetics of clot formation using rotational thromboelastometry. Clotting was initiated by recalcification (12 mM CaCl_2_ final conc.). Control samples were without any addition. Representative coagulation profiles and records of clot formation rate (CFR) for 6 experiments are presented in **(A)**. **(B–D)** Parameters associated with clotting initiation (CT, clotting time; CFT, clot formation time), propagation (alpha angle; MaxV, maximal velocity of clot formation; MaxV-t, time to reach maximal velocity of clotting), and stabilization (MCF, maximum clot firmness; G, shear modulus strength) were measured. Data are means ± S.D. from 6 experiments. Parameters value range in control was: CT: 215–667 s; CFT: 79–176 s; Alpha: 64–74°; MaxV: 12–18 mm*100/s; MaxV-t: 351–855 s; MCF: 65–75 mm; G: 936–1,492. *p < 0.05, **p < 0.01, and ***p < 0.001 vs. control; ^#^*p* < 0.05, ^##^*p* < 0.01, and ^###^*p* < 0.001 vs. adrenaline 10 nM + coll. **(E)** Whole blood samples (supplemented with AF488-fibrinogen to visualize fibrin formation) were incubated without any addition (control) or with adrenaline (for 2 min) or rauwolscine (10 μM, for 10 min) + adrenaline (for 2 min). Kinetics of fibrin formation within thrombi, formed under flow (1,000 s^– 1^) on collagen-coated surfaces, was recorded in confocal microscope. The fibrin(ogen)-associated fluorescence was recorded and expressed as% of maximal fluorescence obtained (considered as 100%) vs. time. Data are means ± S.D. from 4 experiments. *p < 0.05 vs. control; ^#^*p* < 0.05 vs. adrenaline 1 nM.

To test whether adrenaline-triggered procoagulant response would affect the kinetics of platelet-fibrin clot formation we performed measurements under quasi-static (thromboelastometry) and flow conditions. The preincubation of whole blood samples with adrenaline (3–10 nM) and collagen (150 ng/ml) provoked the significant augmentation of the coagulation profile evaluated by thromboelastometry (Figure 6A), which was manifested in the shortening of CT and CFT (initiation phase of clot formation, Figure 6B), increasing in alpha angle, MaxV, and in the shortening of time to MaxV (propagation of clotting, Figure 6C), as well as in a rise in MCF and G (stabilization phase, Figure 6D). These effects were counteracted by the preincubation of samples with rauwolscine (Figures 6A–D).

Under flow conditions (shear 1,000 s^–1^), the presence of adrenaline (1 nM) in whole blood samples was associated with the shortening of time required to start of fibrin appearance within thrombi formed onto collagen, and with substantial increase in fibrin formation rate, both abolished by rauwolscine (Figure 6E).

### Alteration of Platelet-Fibrin Clot Structure and Attenuation of Fibrinolysis by Adrenaline

**FIGURE 7:**
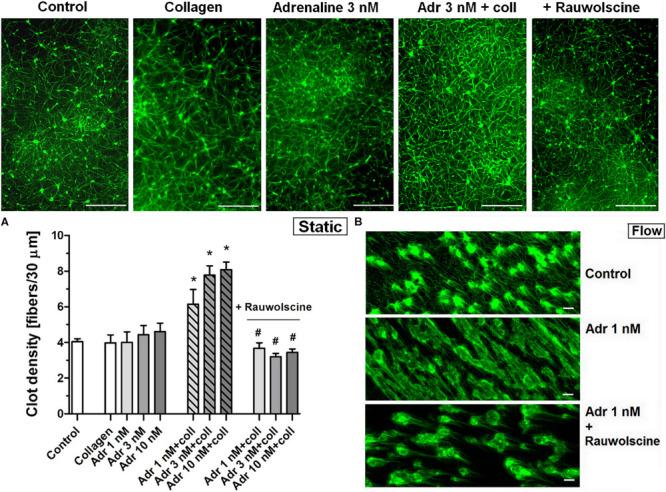
Effect of adrenaline on the architecture of platelet-fibrin clots under static and flow conditions. **(A)** PRP samples were incubated without any additions (control) or with or without rauwolscine (10 μM, for 10 min) followed by the addition of adrenaline (1–10 nM) + collagen (150 ng/ml). Afterward, samples were supplemented with AF488-fibrinogen (to visualize fibrin formation). Clotting was triggered by recalcification (18 mM CaCl_2_, final conc.) and resulted platelet-fibrin clots were analyzed under confocal microscope toward fibrin density. Structures representative for 7 experiments are presented in **(A)**. Bars are means ± S.D. from 7 experiments. *p < 0.01 vs. control; ^#^*p* < 0.01 vs. appropriate samples containing adrenaline and collagen. **(B)** Whole blood samples (supplemented with AF488-fibrinogen) were incubated without any addition (control) or with adrenaline (1 nM, for 2 min) or rauwolscine (10 μM, for 10 min) + adrenaline (for 2 min). Architecture of fibrin mesh within thrombi, formed under flow (1,000 s^– 1^) on collagen, was recorded in confocal microscope. Pictures are representative for 4 experiments. White bar is 30 μm in **(A)** and 10 μm in **(B)**.

To check whether the adrenaline-related augmentation of platelet procoagulant response may exert impact on clot architecture we analyzed the platelet-fibrin clots formed under static and flow conditions using confocal microscopy. The preincubation of PRP samples with adrenaline (1–10 nM) and collagen (150 ng/ml) resulted in a significantly higher density of platelet-fibrin clots formed under static conditions by recalcification (Figure 7A). The presence of adrenaline (1 nM) in whole blood samples resulted in formation of clots on collagen under flow conditions (shear 1,000 s^–1^) reminding more sponge-like mesh than distinctly separated fibers, typically observed in control samples (Figure 7B). Above effects of adrenaline on clots structure were abolished by rauwolscine.

**FIGURE 8:**
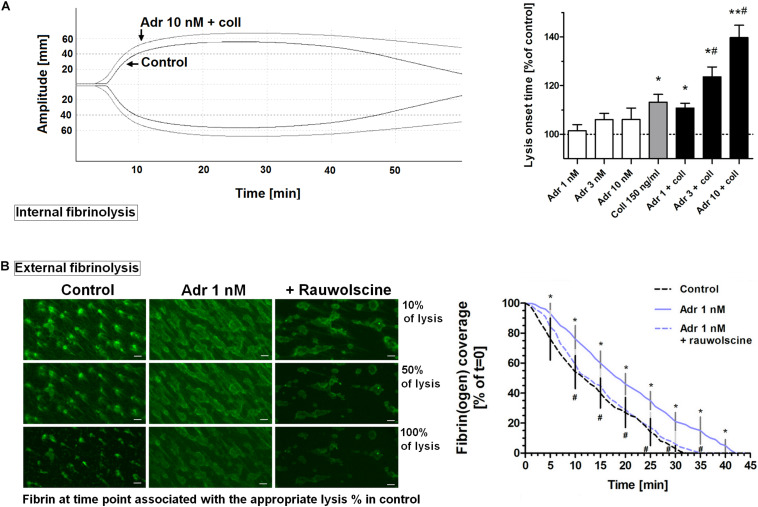
Effect of adrenaline on the kinetics of fibrinolysis. **(A)** Whole blood samples were supplemented with adrenaline (1–10 nM), collagen (150 ng/ml), or their combinations. After 10 min of incubation samples were mixed with tissue factor (100 ng/ml) and tPA (120 ng/ml) and kinetics of internal fibrinolysis [expressed as lysis onset time (LOT)] was evaluated by thromboelastometry. Presented records are representative for 6 experiments. LOT values in controls were in a range of 3,338–4,158 s. Bars are means ± S.D. from 6 experiments. *p < 0.05, **p < 0.01 vs. control; ^#^*p* < 0.05 vs. collagen. **(B)** Whole blood samples were incubated without any additions (control) or with or without rauwolscine (10 μM, for 10 min) followed by the addition of adrenaline (1 nM, for 2 min). After addition of AF488-fibrinogen samples were perfused over collagen-coated surfaces at shear rate 1,000 s^– 1^. After formation of fibrin, clots were washed with a buffer containing tPA (40 nM) and sets of confocal pictures were collected until fibrin-related fluorescent signal decreased to the background level (reflecting progress of external fibrinolysis). Representative pictures of fluorescent fibrin at time points associated with the 10, 50, and 100% of lysis (related to control samples) and kinetics of fibrinolysis are presented. “*t* = 0” means fibrin(ogen)-related fluorescence at start point of fibrinolysis (considered as 100%). Values range in control samples were: to obtain 50% of lysis–407–1,103 s, to 100% of lysis–1,575–2,508 s. *p < 0.05 vs. control; ^#^*p* < 0.05 vs. adrenaline. White bar = 10 μm.

To assess whether adrenaline may modulate the kinetics of fibrinolysis, we measured the rate of lysis under quasi-static (internal fibrinolysis) and flow conditions (external fibrinolysis). In whole blood, the combination of adrenaline (1–10 nM) and collagen (150 ng/ml) produced the adrenaline concentration-dependent elongation of (tPA-triggered) lysis onset time measured by thromboelastometry (Figure 8A). Under flow conditions (shear 1,000 s^–1^), the presence of adrenaline (1 nM) in whole blood samples resulted in the formation of platelet-fibrin clots onto collagen, which were more resistant to tPA-evoked lysis compared to control clots. This was manifested in the significant diminishing of lysis rate which was normalized by rauwolscine (Figure 8B). It is worth to note that the lysis rates observed in this study were notably lower compared to results obtained by Whyte et al. in a similar experimental setup (Whyte et al., 2015). However, while Whyte et al. measured fibrinolysis progress in the presence of tPA inside the platelet-fibrin thrombi, we concentrated on solely “external” fibrinolysis where plasminogen activator was present in a buffer used to wash fully developed thrombi from the outside. Under such conditions fibrin degradation was prolonged due to necessity of the fibrinolytic factors to diffuse inside the fibrin net which can be additionally reduced by the elevated degree of thrombus contraction and fibrin density (both enhanced in adrenaline-treated samples—Figures 2, 7).

## Discussion

In this report we present experimental evidence that clinically relevant adrenaline concentrations may augment platelet-dependent hemostasis in human blood. The flow-based assays used in this study allow for investigating the effect of adrenaline on hemostasis under flow—which is problematic using *in vivo* thrombosis models due to a severe hemodynamic effect of the hormone.

### Effect of Adrenaline on Early Platelet Responses

We found that adrenaline at a concentration as low as 1 nM is able to augment platelet deposition on collagen at shear rate of 1,600 s^–1^ but not at 1,000 s^–1^. This discrepancy is quite unexpected, since Eriksson and Whiss showed an increased accumulation of adrenaline-treated platelets on collagen under static conditions (Eriksson and Whiss, 2013). However, while authors measured the activity of acid phosphatase originated from collagen-bound platelets as a marker of adhesion (such evaluated adhesion is directly associated with a number of platelets attached to collagen), our approach comprised the estimation of the surface area coverage by thrombi as a function of adhesion under flow. In this case, measurements are sensitive to the alterations in thrombi morphology, such as a degree of thrombus consolidation. Since platelet thrombi undergo retraction, which may markedly change their size and morphology (Ono et al., 2008; Tutwiler et al., 2016), we further investigated whether the lack of visible effect of adrenaline on platelet adhesion at shear rate of 1,000 s^–1^ may be connected with its impact on platelet-mediated retraction.

A careful analysis of the pictures of thrombi formed in the presence of adrenaline indicated a higher degree of their consolidation compared to control thrombi. Thrombus/clot contraction measurements performed under flow and static conditions revealed a significant increase in clot retraction rate in adrenaline-supplemented samples. This observation suggests that adrenaline may contribute to a stronger contraction of platelet thrombus which—in specific conditions—may in turn mask the potential enhancement of adhesion, observed by others. On the other hand, augmented adhesion observed under elevated shear rate (1,600 s^–1^) suggests that under specific rheological conditions adrenaline may enhance platelet adhesion stronger than retraction. This is in line with the study of Mustonen and Lassila, where authors showed augmented thrombus formation on collagen at a shear rate of 1,600 s^–1^ in the presence of 10 nM adrenaline (Mustonen and Lassila, 1996).

### Effect of Adrenaline on Platelet Procoagulant Response and Coagulation Kinetics

Here we present, for the first time, direct evidence that clinically relevant concentrations of adrenaline (1–10 nM) are able to significantly augment PS exposure on human platelets exposed to subthreshold collagen. It is worth to notice donor-dependent variation in the cytometric measurements of platelet PS exposure presented here. One possible explanation could be individual differences in platelet reactivity toward adrenaline, associated with adrenergic receptors defect or deficiencies, described previously by other authors in the context of platelet aggregation (Tamponi et al., 1987; Rao et al., 1988; Lindkvist et al., 2019). Adrenaline had enhanced PS exposure on collagen-adhered platelets more strongly at an elevated shear rate which suggests that adrenaline may augment not only platelets deposition on the adhesive surface, but also platelet-related procoagulant response in a shear-dependent manner.

Under flow conditions, where the activity of locally generated thrombin was not inhibited, we observed a temporal increase in the population of PS-exposing platelets which was stronger in adrenaline-treated samples compared to controls. This might be explained by the diffusion of thrombin from sites of initial generation (i.e., thrombus core, rich in procoagulant platelets) to the thrombus periphery and hence in a secondary activation of platelets previously exposed to primal, thrombi-related agonists like thromboxane A_2_ and ADP, but initially not exposing PS (Agbani et al., 2017).

Our examinations of the kinetics of platelet-fibrin clots formation under quasi-static (thromboelastometry) and flow conditions confirmed elevated procoagulant potential in the presence of adrenaline, leading to the augmentation of fibrin formation. Specifically, in thromboelastometric measurements, we observed the shortening of clotting time and the increased alpha angle and maximal velocity of clotting, ROTEM variables that have been found to reflect the rate of thrombin generation (Sørensen et al., 2003). This is consistent with *ex vivo* measurements by Kral et al. (1971), who observed shortened clotting time in whole blood collected from healthy volunteers after adrenaline injection. Correspondingly, under flow, the adrenaline treatment resulted in a notably faster formation of fibrin within platelet-rich thrombi. However, in experiments performed under flow we were unable to test a concentration of adrenaline higher than 1 nM due to the repeated occlusion of the flow chamber. Considering that all of the above described effects were diminished by rauwolscine, an adrenergic receptor antagonist, we conclude that the augmentation of platelet procoagulant response observed here is related directly with the impact of adrenaline on platelet adrenergic receptors.

### Effect of Adrenaline on Clot Structure and Fibrinolysis

Platelet-fibrin clots formed in the presence of adrenaline and low collagen are more resistant to internal and external fibrinolysis. Platelets participate in a phenomenon term “platelet-dependent fibrinolysis resistance” via platelet-dependent procoagulant response leading to augmented coagulation and the development of dense, lysis-resistant fibrin structure (Weisel, 2007; Wolberg, 2007), and by providing retraction of clot which makes it less permeable to external fibrinolytic factors (Tutwiler et al., 2019). Since clot retraction, fibrin formation, and clot density seem to be increased in the presence of adrenaline, we conclude that clinically relevant adrenaline concentrations may participate in the inhibition of fibrinolysis in human blood via augmentation of platelet-related fibrinolysis resistance.

The impact of adrenaline on fibrinolytic system in humans has been investigated in several studies where adrenaline was injected to healthy volunteers (Biggs et al., 1947; Kral et al., 1971; Chandler et al., 1992). The authors observed increased fibrinolytic activity assessed in platelet-depleted plasma, which was most likely connected with elevated tissue plasminogen activator, released from endothelial cells in response to adrenaline. Yet, while these studies considered fibrinolytic activity only in plasma, the precise effect of adrenaline on fibrinolysis *in vivo*, considering the role of platelets, is an important subject for future studies.

### How Much Does This Study Adhere to the Clinical Reality?

The results presented in this paper suggest that clinically relevant adrenaline concentrations, especially combined with low concentrations of collagen, may exert a more complex effect on hemostasis in human blood than it has been considered so far.

The observation that adrenaline-treated platelets are more prone to adhere to collagen and to expose PS at an elevated shear rate is important, since in the circulatory system adrenaline primarily acts as a vasoconstrictor producing an increase in blood flow and hence in shear rate value (Bühler et al., 1983; Bolli et al., 1984). Therefore, its effect on platelet accumulation and platelet-related thrombin generation *in vivo* is likely to be bidirectional, i.e., the augmentation of platelet activation (direct effect) and the creation of hemodynamic condition which additionally predisposes platelets to adhesion and PS exposure. One can hypothesize that this “adrenaline effect” might be more significant in case of arterial thrombosis, where thrombus formation is triggered predominantly via exposure of collagen-rich subendothelium, and the resulting thrombi are composed—in contrast to venous thrombi—mainly of platelets and fibrin (Chernysh et al., 2020). These findings indicate that adrenaline-related effects on platelets are more likely to manifest themselves at pathological shear rates, e.g., in the proximity of atherosclerotic plaques or arterial stents, but also during rapid, adrenaline-evoked vasoconstriction leading to an elevated risk of atherothrombosis. On the other hands, adrenaline supplementation has been proposed as a mean to restore platelet aggregability in ticagrelor (P2Y_12_ antagonist)-receiving patients, thus reducing the perioperative bleeding risk. Singh et al. observed such effect *in vitro* in the presence of 770 nM adrenaline (Singh et al., 2018) and *ex vivo*, where adrenaline infusion at doses as low as 0.15 μg/kg/min (leading to ∼20 nM of plasma adrenaline concentration) moderately improved ADP-induced platelet aggregation and clot formation in the blood collected from healthy volunteers receiving ticagrelor, without a deleterious impact on heart rate and blood pressure (Singh et al., 2019). This effect was associated with the restoration of P2Y_12_-like signaling by adrenaline since P2Y_12_ and α_2_-AR receptors share similar effectors: Gα-mediated inhibition of AC and Gβγ-related activation of PI-3K (Martin et al., 2020). Also other α_2_-AR agonists: noradrenaline and brimonidine have been recently found to restore aggregation in ticagrelor-treated platelets (Bonete et al., 2020). However, above studies did not include evaluation of platelet procoagulant response and fibrinolysis after adrenaline (or other α_2_-AR agonists) infusion.

Our results also show that PS exposure evoked by adrenaline and collagen may be efficiently reduced by agents representing the most commonly used antiplatelet strategies, i.e., cyclooxygenase inhibitor, GPIIb/IIIa blocker, and P2Y_12_ antagonist. Classical antiplatelet therapeutics are therefore likely to show beneficial properties not only in reducing platelet aggregation but also in normalizing the prothrombotic state evoked by a clinically relevant adrenaline concentrations in combination with collagen.

## Conclusion

In human blood clinically relevant concentrations of adrenaline may augment thrombus formation and platelet PS exposure, leading to elevated fibrin formation, altered clot structure, and attenuated fibrinolysis. In case of therapies involving infusion of adrenaline, its impact on other aspects of hemostasis—beside hitherto investigated platelet aggregation and clot formation—especially on platelet procoagulant response and fibrinolysis should be considered. Further studies on the molecular mechanism of adrenaline-triggered platelet activation are in prospect.

## Data Availability Statement

The raw data supporting the conclusions of this article will be made available by the authors, without undue reservation.

## Ethics Statement

The studies involving human participants were reviewed and approved by the Bioethics Committee (Medical University of Bialystok). The patients/participants provided their written informed consent to participate in this study.

## Author Contributions

AG and TM designed research, performed experiments, analyzed the data, and wrote the manuscript. NM performed experiments and analyzed the data. EC and TR analyzed the data. All authors reviewed and edited the manuscript and approved the final version of the manuscript.

## Conflict of Interest

The authors declare that the research was conducted in the absence of any commercial or financial relationships that could be construed as a potential conflict of interest.

## Supplementary Material

Supplementary Figure 1 Experimental setup for flow experiments. Wall shear rate was generated by perfusion of whole blood through flow chamber at defined flow rate (Q). Platelet thrombus or platelet-fibrin clot was formed on collagen-coated surface dependent on the presence or absence of direct thrombin inhibitor (e.g., PPACK). Real-time or end-stage observations were conducted using confocal microscope.

## Abbreviations

AC: adenylate cyclase
ADP: adenosine diphosphate
AF: Alexa Fluor
AR: adrenergic receptor
ASA: acetylsalicic acid
DiO (3-octadecyl-2-[3-(3-octadecyl-2(3H)-benzoxazolylidene)-1- propenyl]-: perchlorate
FITC: fluorescein isothiocyanate
LOT: lysis onset time
PI3-K: phosphoinositide-3 kinase
PS: phosphatidylserine
PE: phycoerythrin
RBC: red blood cells (erythrocytes)
ROTEM: rotational thromboelastometry
tPA: tissue plasminogen activator.
