# Supplementary figures and images for: Adrenaline May Contribute to Prothrombotic Condition via Augmentation of Platelet Procoagulant Response, Enhancement of Fibrin Formation, and Attenuation of Fibrinolysis
Source: Front Physiol. 2021 May 5;12:657881. doi: 10.3389/fphys.2021.657881 (PMC8134743; doi:10.3389/fphys.2021.657881)

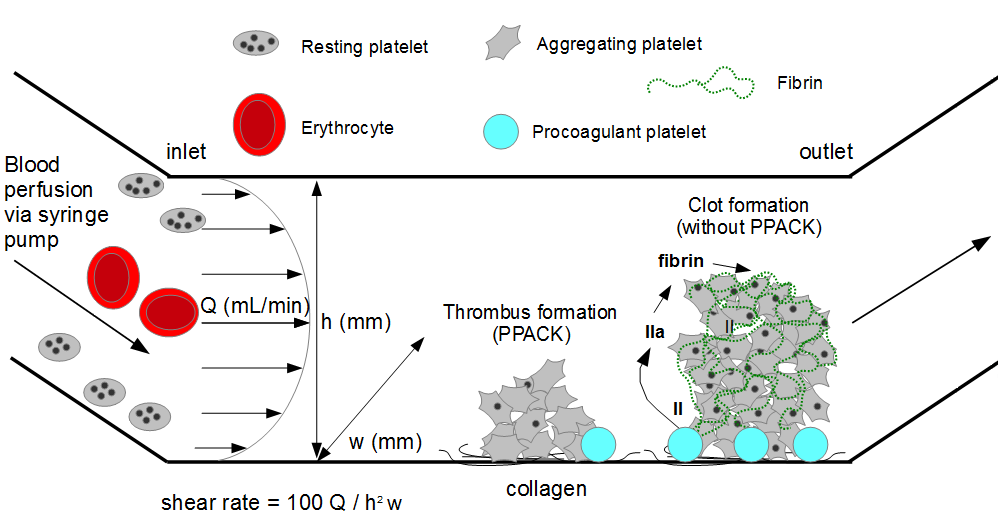

Supplement: Supplementary Figure 1 — Experimental setup for flow experiments. Wall shear rate was generated by perfusion of whole blood through flow chamber at defined flow rate (Q). Platelet thrombus or platelet-fibrin clot was formed on collagen-coated surface dependent on the presence or absence of direct thrombin inhibitor (e.g., PPACK). Real-time or end-stage observations were conducted using confocal microscope. [file Image_1.TIF]
